# Choroidal and peripapillary changes in high myopic eyes with Stickler syndrome

**DOI:** 10.1186/s12886-020-01777-3

**Published:** 2021-01-04

**Authors:** Olivia Xerri, Federico Bernabei, Elise Philippakis, Cyril Burin-Des-Roziers, Pierre-Olivier Barale, Olivier Laplace, Claire Monin, Dominique Bremond-Gignac, Gilles Guerrier, Sophie Valleix, Antoine Brezin, Pierre-Raphaël Rothschild

**Affiliations:** 1 Service d’Ophtalmologie, Hôpital Necker-Enfants Malades, AP-HP, F-75014 Paris, France; 2 Service d’Ophtalmologie, Ophtalmopôle de Paris, Hôpital Cochin, AP-HP, 27 rue du Faubourg Saint Jacques, 75014 Paris, France; 3 Service d’Ophtalmologie, Hôpital Lariboisière, AP-HP, F-75014 Paris, France; 4 Université de Paris, Centre de Recherche des Cordeliers, INSERM, UMR_1138, F-75006 Paris, France; 5 Service d’Ophtalmologie, Hôpital des Quinze-Vingts, Paris, France; 6 Anaesthetic and Intensive Care Department, Hôpital Cochin, Paris Descartes university, 75014 Paris, France; 7 Laboratoire de Génétique Moléculaire, Faculté de Médecine Paris, Hôpital Necker-Enfants Malades, Université de Paris, AP-HP; Inserm, U_1163, Institut IMAGINE, F-75014 Paris, France

**Keywords:** Choroidal thickness, Congenital myopia, Hereditary vitreopathy, High myopia, Stickler syndrome

## Abstract

**Background:**

To compare different clinical and Spectral-Domain Optical Coherence Tomography (SD-OCT) features of high myopic eyes with Stickler syndrome (STL) with matched controls.

**Methods:**

Patients with genetically confirmed STL with axial length ≥ 26 mm and controls matched for axial length were included. The following data were obtained from SD-OCT scans and fundus photography: choroidal and retinal thickness (respectively, CT and RT), peripapillary atrophy area (PAA), presence of posterior staphyloma (PS).

**Results:**

Twenty-six eyes of 17 patients with STL and 25 eyes of 19 controls were evaluated. Compared with controls, patients with STL showed a greater CT subfoveally, at 1000 μm from the fovea at both nasal and temporal location, and at 2000 and 3000 μm from the fovea in nasal location (respectively, 188.7±72.8 vs 126.0±88.7 μm, 172.5±77.7 vs 119.3±80.6 μm, 190.1±71.9 vs 134.9±79.7 μm, 141.3±56.0 vs 98.1±68.5 μm, and 110.9±51.0 vs 67.6±50.7 μm, always *P*< 0.05). Furthermore, patients with STL showed a lower prevalence of PS (11.5% vs 68%, *P*< 0.001) and a lower PAA (2.2±2.1 vs 5.4±5.8 mm^2^, *P*=0.03), compared with controls.

**Conclusions:**

This study shows that high myopic patients with STL show a greater CT, a lower PAA and a lower prevalence of PS, compared with controls matched for axial length. These findings could be relevant for the development and progression of myopic maculopathy in patients with STL.

## Background

Stickler syndrome (STL) is an inherited connective tissue disorder, that leads to a broad spectrum of manifestations including facial, skeletal, ear, and ocular abnormalities [1, 2]. The disease has an estimated incidence of 1: 7.500 to 9.000 newborns and is caused by mutations in the genes encoding for different types of collagen, namely II, IX, and XI [3–6]. The most common pathogenic variants associated with STL are found in the *COL2A1* and *COL11A1* gene, that account respectively for the 80–90% and 10–20% of cases [7]. Common ocular findings include congenital myopia, vitreous abnormalities, and early onset cataract [2]. Furthermore, the disease represents a serious sight-threating condition, due to a high risk of developing retinal detachment that seems to be related to an abnormal vitreoretinal interface as well as a complication of myopia [8, 9].

Few studies have reported the prevalence of high myopia (HM) in STL, and it is estimated to occur in 76 to 80% of patients [8, 10]. Moreover HM is typically present at birth and has a non-progressive course [11]. This is a highly distinctive feature of the disease, because it is often the only manifestation during infancy. In idiopathic HM the elongation of the axial length and the progressive increase in the curvature of the posterior pole occurs later in life and are associated with the development of sight-threatening chorioretinal complications including choroidal neovascularization (CNV), myopic traction maculopathy and macular hole [12, 13]. To date, except for retinal detachment, no other severe complications related to HM have been described in patients with STL. The improvements in spectral domain - optical coherence tomography (SD-OCT) technology, such as enhanced-depth imaging (EDI) mode, allow a better visualization of retinal and choroidal structures along with a more precise characterization of quantitative parameters such as choroidal and retinal thickness (respectively, CT and RT), [14]. Thus, the aim of this study was to investigate clinical and SD-OCT characteristics of highly myopic eyes in patients with genetically confirmed STL and to compare them with those of highly myopic patients without STL.

## Methods

### Design and patients

This retrospective observational study was conducted in the setting of a collaborative project named French vitreoretinopathy study group (FVSG). Patients were identified from databases of the retina service of different tertiary eye care centers and were subsequently examined at least once by one of the authors (PRR) at the OphtalmoPole de Paris, Hôpital Cochin (Paris, France). Referring hospitals included: Hôpital des Quinze-Vingts (Paris, France), Hôpital Lariboisière, (Paris, France) and Hôpital Necker-Enfants Malades, (Paris, France). Institutional review board approvals for retrospective chart reviews were obtained commensurate with the respective institutional requirements prior to the beginning of the study. Described research was approved by the ethics committee of the French society of ophthalmology and adhered to the tenets of the declaration of Helsinki. Fully written informed consent was obtained for all patients. Patients with genetically confirmed STL were identified at the retina service of the participating centers and those with HM, defined by the presence of an axial length of 26 mm or longer, were included in the study group. Subjects with HM, not suspected to have SLT based on family history and on the absence of ocular and extraocular features, were matched for axial length and included as controls. Exclusion criteria for both groups were as follows: history of retinal detachment and other retinal diseases, glaucoma, any previous retinal laser photocoagulation or surgical procedure except for cataract surgery and missing data from medical records. Furthermore, in order to evaluate the status of the retina and the choroid in the absence of macular complications related to myopia, eyes with lacquer cracks, myopic CNV, myopic traction maculopathy, dome-shaped macula and macular hole were excluded from both groups.

### Data collection

The following data were extrapolated from medical records: age, sex, axial length (IOL Master700®, Carl Zeiss Meditec, Jena, Germany), lens status, fundus photography of the posterior pole encompassing optic nerve and macula (Canon CR2 plus AF®, Canon, Tokyo, Japan and/or Optos® California, Optos, Marlborough, MA, USA), SD-OCT scans and infra-red (IR), fundus images (Spectralis®, Heidelberg Engineering Inc., Heidelberg, Germany).

SD-OCT horizontal 30° line scan, passing through the fovea, were acquired with EDI and high-resolution mode, obtaining an average of 60 scans with a quality rate superior to 25. On SD-OCT scan, CT was defined as the vertical distance from the hyperreflective line of the Bruch’s membrane to the hyperreflective line of the inner surface of the sclera. CT at the sub-foveal location and at 1000, 2000 and 3000 μm from the fovea, in the nasal and temporal locations were measured.

On SD-OCT scan, total RT was defined as the vertical distance from the hyperreflective line of the vitreoretinal interface to the hyperreflective line of the retinal pigment epithelium. RT at the fovea, at the nasal and temporal clivus and at 3000 μm from the fovea in the nasal and temporal locations were measured. The peripapillary atrophy area (PPA) was obtained using a previously described modified technique [15]. In brief, the optic nerve area was subtracted to the area bounded by the edge of atrophy based on IR images [15]. All the measurements were performed independently by two ophthalmologists (OX and FB), both blinded to patient’s characteristics, by using the built-in calipers of the software. The average of the 2 measurements was used for analysis.

The presence and the location of posterior staphyloma (PS) were evaluated by both fundus photography and SD-OCT scan and classified as follows: 0) absence of PS, i) PS with macular involvement, ii) PS without macular involvement, iii) other [16].

### Statistical analysis

Data analysis was conducted with XLSTAT Version 2017.02.43358 (Addinsoft, Paris, France). Quantitative data are expressed as mean ± standard deviation (SD) and qualitative data are expressed as percentages, with a confidence interval of 95% [CI 95%]. The Shapiro-Wilk’s test was used to assess normality of data. An independent sample t-test was used to compare normally distributed variables between two groups, while Mann-Whitney U test was used for not normally distributed variables. Qualitative variables were compared between the two groups using chi-square test or Fisher exact test when necessary. The correlations of sub-foveal CT with age, axial length, foveal RT, and PPA, and of PPA with age and axial length were examined using Pearson’s correlation analysis. A *P* value < 0.05 was considered statistically significant.

## Results

**Fig. 1:**
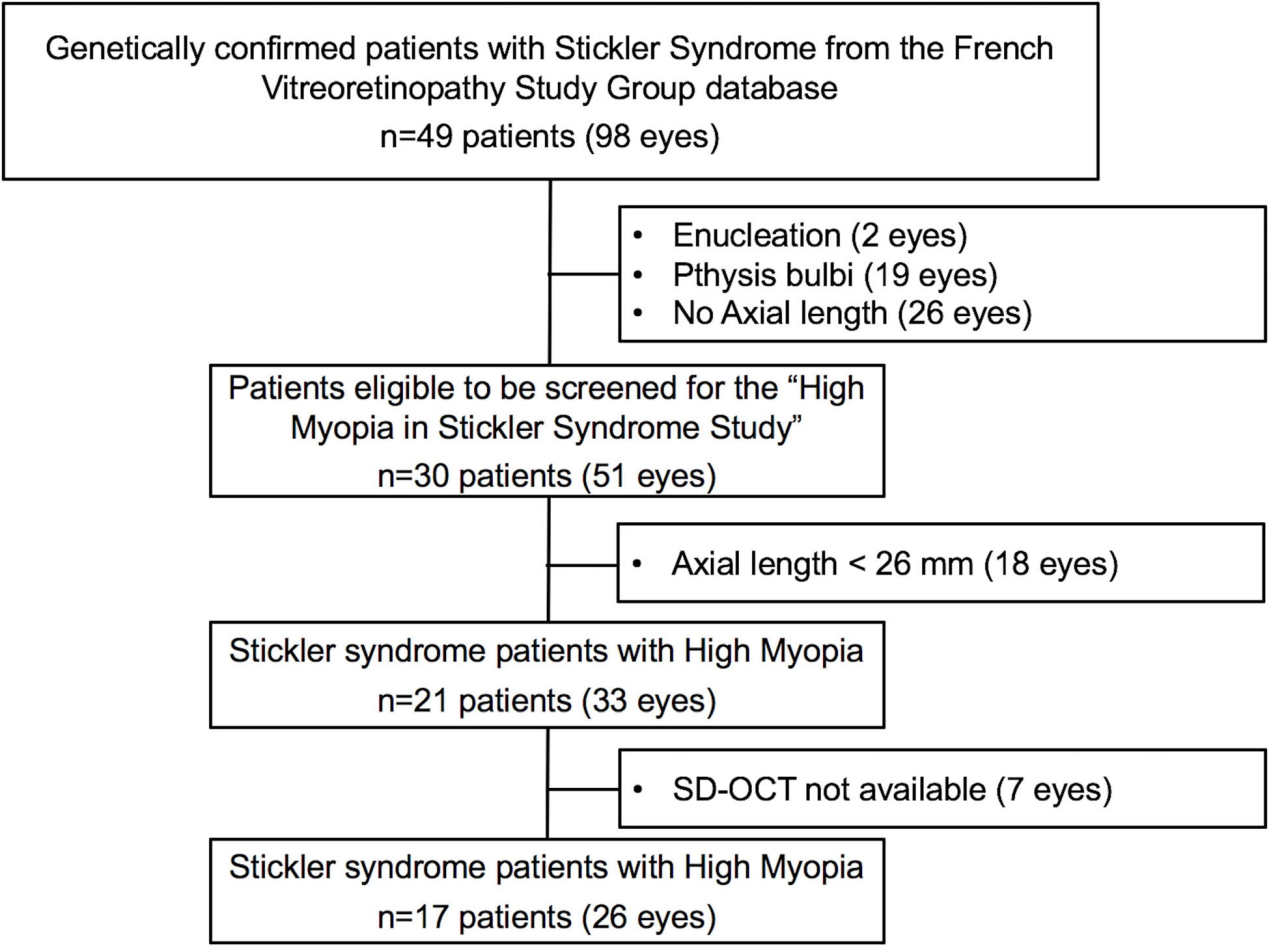
Inclusion flow chart of patients with Stickler syndrome and high myopia

Forty-nine patients (98 eyes) with genetically confirmed STL were identified from the FVSG database. All the cases a had a positive family history for STL and all had a pathogenic mutation in the *COL2A1* gene. Forty-seven eyes were excluded for the following reasons: enucleation (*n* = 2), phthisis bulbi (*n* = 19), absence of axial length measurement (*n* = 26). Of the remaining 30 patients (51 eyes), 21 (70.0%, [56.6–86.4]) (33 eyes) presented HM. Seven eyes were subsequently excluded from this cohort for the lack of SD-OCT scans. Finally, 26 eyes of 17 patients with HM and genetically confirmed SLT were included (STL group). The flow chart of the inclusion process is represented in Fig. 1.

The demographic and clinical characteristics of patients with STL and control subjects are reported in Table 1.

**Table 1:** Demographic and clinical characteristics of patients with stickler syndrome and control subjects

Characteristics	Stickler group	Control group	*P*
Patients (n)	17	19	
Eyes (n)	26	25	
Phakic (n)	11	18	
Sex (m/f)	7/10	11/8	0.3
Age (years ± SD)	34.5 ±13	39.5 ±11.1	0.16
Axial Length (mm ± SD)	28.0 ± 2.0	29.2 ± 2.5	0.07

*SD* Standard deviation

No significant differences were found in axial length between the two groups (*P* > 0.05). A significantly lower prevalence of PS was found in STL group compared with control group (*P* < 0.01). In particular, PS was present in 3 eyes (11.5% [0–23.8]) of the STL group and in 17 eyes (68.0% [49.7–86.3]) of the control group. In the 3 eyes of STL group PS involved the macula, while in the control group, 13 eyes presented PS that involved the macula, and in 4 eyes PS involved the peripapillary region.

The choroidal and retinal parameters of patients with STL and control subjects are reported in Table 2.

**Table 2:** Choroidal and retinal parameters of patients with stickler syndrome and control subjects

Parameter	Stickler group	Control group	*P*
Choroidal Thickness (μm)			
Sub-foveal	188.7 ± 72.8	126.0 ± 88.7	**0.01**
Nasal 1000^a^	172.5 ± 77.7	119.3 ± 80.6	**0.03**
Nasal 2000	141.3 ± 56.0	98.1 ± 68.5	**0.02**
Nasal 3000	110.9 ± 51.0	67.6 ± 50.7	**0.01**
Temporal 1000	190.1 ± 71.9	134.9 ± 79.7	**0.03**
Temporal 2000	182.8 ± 61.3	140.8 ± 76.0	0.06
Temporal 3000	186.0 ± 62.6	142.9 ± 78.5	0.08
Total Retinal Thickness (μm)			
Foveal	224.5 ± 43.9	230.9 ± 36.8	0.26
Nasal clivus	313.4 ± 68.8	325.6 ± 42.8	0.82
Nasal 3000	265.9 ± 41.4	264.8 ± 50.1	0.93
Temporal clivus	299.8 ± 62.0	318.5 ± 36.2	0.42
Temporal 3000	237.0 ± 38.6	244.8 ± 32.0	0.76
Peripapillary atrophy (mm^2^)	2.2 ± 2.1	5.4 ± 5.8	**0.03**
Posterior Staphyloma (n (%))	3 (11.5% [0–23.8])	17 (68.0% [49.7–86.3])	**< 0.001**

*SD* Standard deviation. ^a^ Denotes the position 1000 μm nasal to the fovea. The same naming convention is used for the subsequent entries. Significant *P* values (< 0.05) are in bold

**Fig. 2:**
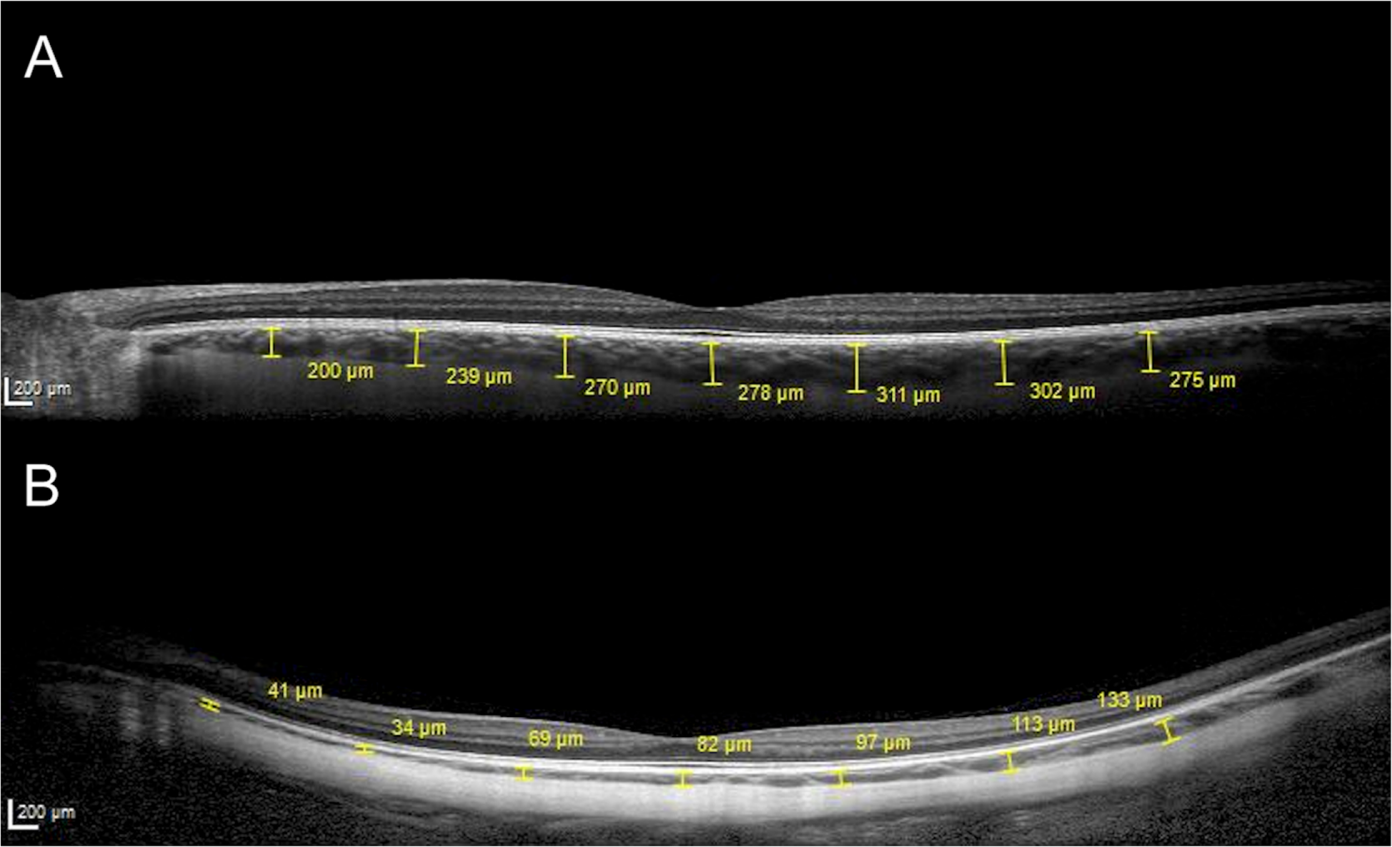
Representative macular SD-OCT scans of a 36 years old male with Stickler syndrome, presenting an axial length of 27.3 mm (**a**) and of 35 years old male with high myopia, presenting an axial length of 28.1 and posterior staphyloma (**b**). In both patients, choroidal thickness measurements were performed at sub-foveal location and at 1000, 2000 and 3000 μm from the fovea, in the nasal and temporal locations

In brief, mean CT at the sub-foveal location was higher in patients with STL compared to control subjects (188.7 ± 72.8 vs. 126.0 ± 88.7 μm, *P* = 0.01) (Fig. 2).

**Fig. 3:**
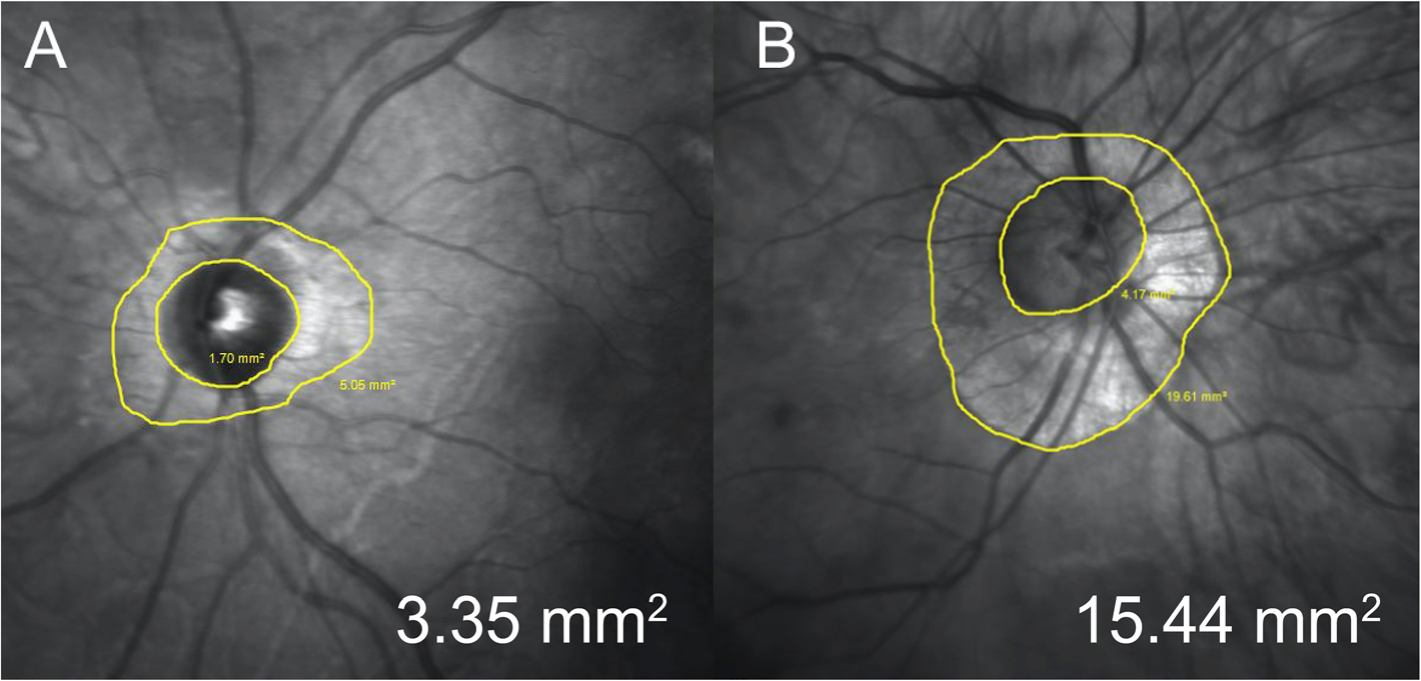
Representative infra-red optic nerve images of 34 years old female with Stickler syndrome (**a**) and of 33 years old female enrolled as control (**b**). Peripapillary atrophy area were respectively 3.35 mm^2^ and 15.44 mm^2^

Moreover, significantly higher values of mean CT measured at 1000 μm from the fovea at both nasal and temporal location and at 2000 and 3000 μm from the fovea in nasal location, were found in the STL group compared to control group (respectively, 172.5 ± 77.7 vs. 119.3 ± 80.6 μm, *P* = 0.03; 190.1 ± 71.9 vs. 134.9 ± 79.7 μm, *P* = 0,03; 141.3 ± 56 vs. 98.1 ± 68.5 μm, *P* = 0.02 and 110.9 ± 51 vs. 67.6 ± 50.7 μm, *P* = 0.01). Conversely, no significant differences were found between the two groups in mean CT at 2000 and 3000 μm from the fovea in the temporal location and in RT at all measured locations including the fovea, the nasal and temporal clivus and the 3000 μm from the fovea in the nasal and temporal location (all *P* > 0.05). A significantly lower value of mean PPA was found in patients with STL compared to controls (2.2 ± 2.1 vs. 5.4 ± 5.8 mm^2^, *P* = 0.03) (Fig. 3).

In the two groups sub-foveal CT showed a significant correlation with age (*R*= − 0.3, *P* = 0.02), axial length (*R*= − 0.7, *P* < 0.01) and PPA (*R* = − 0.5, *P* < 0.01). In addition, PPA showed a significant correlation with axial length (*R*=0.5, *P* < 0.01).

## Discussion

In the present study we evaluated different SD-OCT parameters and clinical characteristics in high myopic patients with STL and we compare them with controls, matched for axial length. Surprisingly, in the STL group, eyes presented a lower prevalence of PS compared with the control group, that showed values that are in agreement with the literature [17]. In addition, patients with STL presented a significantly greater CT subfoveally and in all the analyzed locations, except for the two more distant from the fovea at the temporal side.

The development of myopia is typically associated with a progressive increase of axial length along with the myopic refractive error [18]. The association of HM with a thin CT has been well established and it seems to be in part related to the increased axial length [19]. However, a recent meta-analysis failed to prove axial length as an independent risk factor for the thinning of the choroid, suggesting the role of other variables in the CT decreasing process [20]. Interestingly, a recent report from the Beijing Eye Study cohort, showed that the presence of PS was the most important factor affecting CT in highly myopic patients [21]. Our results are in agreement with those of Zhou and coauthors, supporting the strong association between these two parameters [21]. In particular, we found that control subjects presented a high prevalence of PS along with a thinner CT, while patients with STL showed a relatively well-preserved choroid and a lower prevalence of PS. In addition, Ellabban and collaborators investigated the CT in eyes with PS involving the macula and found a thinning of the choroid in the whole macular area except for a relatively well-preserved choroid in the temporal area, that seemed to be less prone to be affected by PS [22]. This finding could help explain the absence of difference in CT in the area temporal to the fovea between STL and control group, given that, in almost the totality of patients, PS was localized at the posterior pole.

It is recognized that both PS and a thin choroid are risk factors for the development of complications related to HM [23, 24]. Several studies have indeed reported that the presence of PS and choroidal thinning are associated with a higher rate of diffuse chorioretinal atrophy, CNV and lacquer cracks in eyes with HM and are considered as predictive markers for retinal complications [20, 23–25]. Furthermore, it is now admitted that the presence of PS is associated with the development of myopic macular lesions and that it is a cause of progression of myopic maculopathy rather than a consequence [16].

To the best of our knowledge, no complications related to myopic maculopathy have been described in the setting of STL. The low rate of PS and the relatively well-preserved choroid might help explain the apparent absence of macular complications related to HM in this peculiar subset of patients.

Although there is no proven explanation for the lower rate of PS and the subsequent preservation of the choroid, one of the main differences between STL and non-STL patients resides in the age of onset of the elongation of the eye. Indeed, patients with STL typically born with HM, the so-called “congenital myopia”. Conversely, in non-STL subjects the elongation of the eye occurs several years after birth, commonly at the school age [18]. It can be speculated that the elongation of an immature tissue in utero may result in a better tissue-adaptation. Conversely, in a post-mature state, a worse adaptation could occur, leading to scleral weakness (staphyloma) and to a subsequent choroidal thinning and eventually to complications such as lacquer cracks and CNV.

In line with this hypothesis, we also found that patient with STL presented a lower PPA. Several studies have suggested that PPA could represent a reliable marker for monitoring the progression of HM and it has been shown that PPA is positively correlated with age and axial length [15, 26]. Our results in agreement with those of Liu et al. also showed a correlation between PPA and axial length [15]. No difference was found in RT in all the measured locations between STL and control group. These results are in keeping with a previous study suggesting that RT is not influenced by CT [27].

We acknowledge some limitations in the design of our study. First, a notable number of eyes were excluded for enucleation or phthisis bulbi, presumably due to retinal detachment. It can not be excluded that they may also differ from other STL eyes in posterior pole findings. Second, the small sample size represents other limitation. However, given the rarity of the disease and the stringent inclusion criteria along with the genetic confirmation for all patients with STL, the sample size is considerable. Future studies with larger sample size may warrant further consideration. Finally, the absence of genetic testing in the control group is another limit. However, the exclusion of subjects with family history or any manifestations of STL makes it unlikely that these patients have been included as controls.

## Conclusions

In conclusion, highly myopic patients with STL showed a significantly thicker choroid along with a lower PAA and a lower prevalence of PS compared with controls, after accounting for axial length. Further prospective studies are required to better characterize the features of myopia in patients with STL. These findings help to shed light on the pathogenetic mechanisms underlying pathologic myopia and could be relevant for the development and progression of myopic maculopathy in patients with STL.

## Data Availability

The datasets used and/or analysed during the current study are available from the corresponding author on reasonable request.

## Abbreviations

CT: Choroidal thickness
CNV: Choroidal neovascularization
EDI: Enhanced-depth imaging
FVSG: French vitreoretinopathy study group
HM: High myopia
IR: Infra-red
PPA: Peripapillary atrophy area
PS: Posterior staphyloma
RT: Retinal thickness
SD-OCT: Spectral domain - optical coherence tomography
STL: Stickler syndrome
